# Neurons in the inferior colliculus of the rat show stimulus-specific adaptation for frequency, but not for intensity

**DOI:** 10.1038/srep24114

**Published:** 2016-04-12

**Authors:** Daniel Duque, Xin Wang, Javier Nieto-Diego, Katrin Krumbholz, Manuel S. Malmierca

**Affiliations:** 1 Auditory Neuroscience Laboratory, Institute of Neuroscience of Castilla y León (INCYL), University of Salamanca, Salamanca 37007, Spain; 2 MRC Institute of Hearing Research, University Park, Nottingham, NG7 2RD, UK; 3 Department of Cell Biology and Pathology, Faculty of Medicine, University of Salamanca, Campus Miguel de Unamuno, 37007, Salamanca, Spain; 4 Salamanca Institute for Biomedical Research (IBSAL), Salamanca, Spain

## Abstract

Electrophysiological and psychophysical responses to a low-intensity probe sound tend to be suppressed by a preceding high-intensity adaptor sound. Nevertheless, rare low-intensity deviant sounds presented among frequent high-intensity standard sounds in an intensity oddball paradigm can elicit an electroencephalographic mismatch negativity (MMN) response. This has been taken to suggest that the MMN is a correlate of true change or “deviance” detection. A key question is where in the ascending auditory pathway true deviance sensitivity first emerges. Here, we addressed this question by measuring low-intensity deviant responses from single units in the inferior colliculus (IC) of anesthetized rats. If the IC exhibits true deviance sensitivity to intensity, IC neurons should show enhanced responses to low-intensity deviant sounds presented among high-intensity standards. Contrary to this prediction, deviant responses were only enhanced when the standards and deviants differed in frequency. The results could be explained with a model assuming that IC neurons integrate over multiple frequency-tuned channels and that adaptation occurs within each channel independently. We used an adaptation paradigm with multiple repeated adaptors to measure the tuning widths of these adaption channels in relation to the neurons’ overall tuning widths.

Adaptation refers to the suppression of the brain’s response to repeatedly or frequently occurring sensory stimuli. Adaptation has been found from single-neuron to macroscopic population responses and is ubiquitous across sensory systems^1^,^2^. Nevertheless, its functional role remains debated. For example, it has been suggested that adaptation might enable the brain to efficiently encode stimuli with time-varying statistical properties^3^,^4^. Adaptation tends to be specific to the repeated stimulus and not generalize to other, rare stimuli. This has led to the suggestion that adaptation might facilitate the detection of unexpected *deviant* events in the environment^5^,^6^.

The oddball paradigm involves presentation of rare *deviant* stimuli interspersed among frequent *standard* stimuli. In the auditory domain, deviants elicit an enhancement in electroencephalographic response, referred to as the mismatch negativity (MMN)^7^. The MMN can be elicited with a variety of different deviant features, including sound frequency, duration, complex pitch or sound location^8^,^9^,^10^,^11^. Importantly, an MMN can be elicited by decrements in sound duration^12^,^13^ or sound intensity^14^ and even by sound omissions^15^. This behavior is difficult to explain in terms of recruitment of unadapted neural elements by the deviants and has therefore been interpreted to suggest that the MMN represents a deviance detection process^16^.

Enhanced responses to deviant compared to standard sounds have been observed in individual auditory neurons, both in cortex^17^,^18^ and in subcortical stations^19^,^20^,^21^, and are described as stimulus-specific adaptation (SSA^22^). SSA has been widely proposed as a single-neuron precursor or correlate of the MMN^5^,^17^,^23^. Like the MMN, SSA can be elicited by features other than frequency^24^,^25^. However, it remains unclear whether, or to what extent, SSA reflects true deviance sensitivity as observed in the MMN^22^,^26^, and, in particular, where in the ascending auditory pathway sensitivity to low-intensity deviants embedded in a sequence of high-intensity standards might first emerge^26^,^27^,^28^,^29^.

The current study addresses this question by measuring responses to oddball sequences where the standard and deviant have the same frequency but differ in intensity (referred to as intensity oddball sequences) from single inferior colliculus (IC) neurons in anesthetized rats. For comparison, we also measured responses to frequency- and double (frequency and intensity) oddball sequences. We modelled the measured responses assuming that IC neurons integrate over multiple frequency-tuned channels and that adaptation occurs independently within each of these channels (henceforth referred to as *adaptation channels*^22^,^30^). We devised a novel adaptation paradigm with repeated adaptors (repeated adaptation paradigm, or RAP), which allowed us to characterize the tuning widths of the adaptation channels in relation to the neurons’ overall tuning widths. Our results showed no evidence of SSA for low-intensity deviant sounds in the IC. They indicate that SSA in the IC is only generated when the deviant sound activates different frequency-tuned channels than the standard sound and suggest that, at least in this subcortical relay station, SSA is dependent on input-specific adaptation mechanisms^31^.

## Results

**Figure 1:**
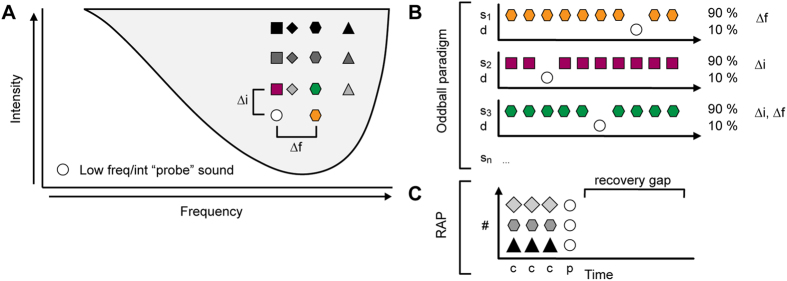
Experimental design. (**A**) Schematic FRA showing the stimuli in the frequency, intensity and double oddball conditions. The lower-frequency/intensity stimulus is shown by the white circle and the different high-frequency/intensity sounds are shown by different colored symbols. (**B**) Schematic representation of frequency (top), intensity (middle) and double (bottom) oddball paradigms. The color and shape of the symbols is as in (**A)**. In this example, the high-frequency/intensity sound is the as standard. (**C)** Schematic representation of the repeated adaptation paradigm (RAP). The adaptor (gray symbols) was presented at a wide range of frequencies and intensities throughout the FRA (white circle; see panel (**A)**).

We recorded oddball paradigm responses from 120 single units in the IC of the rat. We determined the frequency response area of each neuron (FRA) and chose a pair of frequencies, *f*_*1*_ and *f*_*2*_, within the FRA to evaluate SSA in a frequency oddball paradigm (Fig. 1A). Then, in order to test whether SSA is generated by intensity-deviants, we fixed the lower of the two frequencies (*f*_*1*_) and tested oddball paradigms where the standards and deviants differed in intensity only, or in both frequency and intensity (intensity and double oddball paradigms, respectively). In the following, we first describe the responses for the frequency oddball paradigm, where the frequency difference between the standards and deviants, Δ*f*, was 0.1 (Fig. 1B). Second, we describe the intensity and double oddball paradigms with the smallest intensity difference of Δ*i* = 10 dB (Fig. 1B). Then, we present the responses for the intensity and double oddball paradigms with larger intensity differences. Finally, we present the data obtained with the repeated adaptation paradigm (RAP; Fig. 1C; see Methods), which was measured in 33 additional units (not contained within the main sample of the 120 units). We use a model to test if adaptation channels in the IC are determined by the tuning properties of the cochlear frequency channels.

### SSA to frequency and double, but not pure intensity, deviants

**Figure 2:**
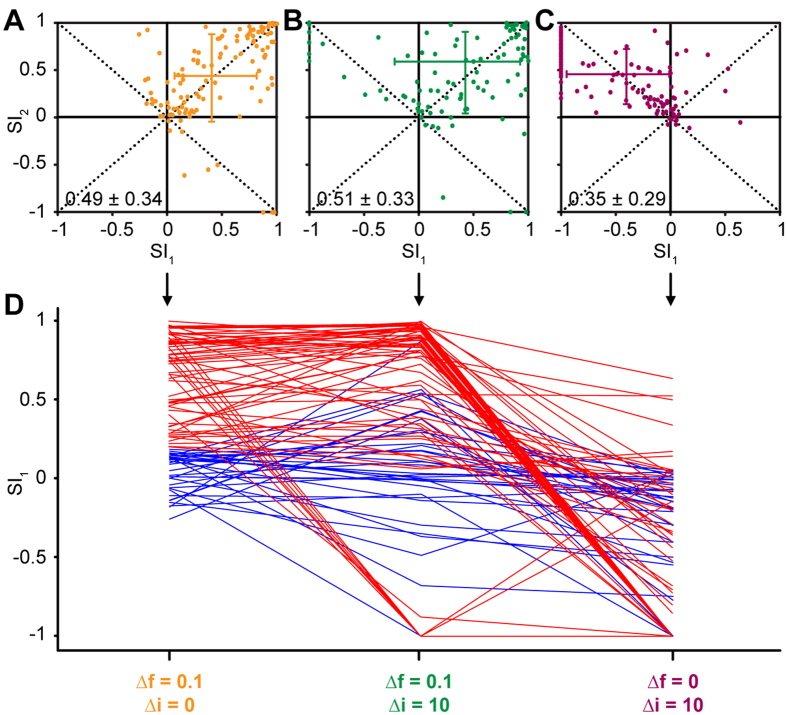
IC neurons do not show intensity SSA. (**A**) Scatter plot of suppression indices (SI) for higher- frequency (SI_2_) versus lower-frequency (SI_1_) deviant stimulus in the frequency oddball paradigm (Δ*f* = 0.1). Each dot represents one neuron. The cross indicates the median and interquartile range for each index. The median CSI value and interquartile range at the bottom of the panel. (**B**) Same as (**A**) but for the double oddball condition with Δ*f* = 0.1 and Δ*i* = 10 dB. (**C**) Same as (**A**,**B**) but for the intensity oddball condition with Δ*i* = 10 dB. (**D**) SI_1_ values for neurons with low (<0.18; blue) or high (≥0.18; red) CSI values in the frequency oddball condition (**A**), plotted across all three oddball conditions (abscissa).

The common SSA index (CSI) was used to quantify SSA in the frequency oddball paradigm (Δ*f* = 0.1, SOA = 250 ms). Across *n* = 120 neurons, the CSI ranged from −0.09 to 0.99 with a mean of 0.49 ± 0.34 (mean ± S.D.), confirming our previous results^19^,^32^,^33^. Based on these CSI values, we defined two populations of neurons, one with strong SSA and the other with weak SSA, by setting a CSI cutoff criterion of +0.18 (the same value as used in previous studies^21^). Based on this criterion, 70% of neurons (*n* = 84) showed strong SSA and 30% (*n* = 36) showed weak SSA. SSA was also quantified using the frequency-specific SSA index (SI_1/2_). As expected based on previous results^19^,^32^,^33^,^34^, in the majority of neurons, both SI values were positive (data points located in the upper right quadrant of Fig. 2A; SI_1_ = 0.42 ± 0.46 (mean ± S.D.); SI_2_ = 0.45 ± 0.38). This indicates that the responses to both frequencies (*f*_1_ and *f*_2_) were more strongly adapted when presented as standard than when presented as deviant, and thus that SSA was present.

Figure 2B shows the SI scatter plot for the double oddball condition where Δ*f* was 0.1 as in the frequency oddball condition, but Δ*i* was 10 dB (see green hexagon in Fig. 1A; measured in *n* = 97 neurons). The CSI values recorded in the double oddball condition ranged from −0.04 to 0.99, with a mean of 0.51 ± 0.33. As for the frequency oddball condition, in the majority of neurons, both SI values were positive (data points in upper right quadrant of Fig. 2B), again indicating SSA (SI_1_ = 0.48 ± 0.43; SI_2_ = 0.36 ± 0.57). However, in a small proportion of neurons, SI_1_ was negative (data points in upper left quadrant), indicating that the lower-frequency/intensity stimulus (*f*_*1*_, *i*_*1*_) elicited a smaller response when presented as deviant than when presented as standard.

Figure 2C shows the SI scatter plot for the intensity oddball paradigm (Δ*f* = 0) with the same intensity difference as in the previous two conditions (Δ*i* = 10 dB; *n* = 117 neurons). In this condition, the CSI values ranged from −0.04 to 0.92 with a mean of 0.35 ± 0.29. Note that, whilst the CSI values for the intensity oddball condition were smaller than for the frequency and double oddball conditions (Kruskall-Wallis ANOVA on Ranks test; *H* = 16.70; *p* < 0.001), the mean CSI value was still positive. This would seem to imply that the intensity oddball condition also elicited SSA. However, the SI scatter plot shows that, in the majority of neurons (*n* = 95, or 81%), SI_1_ was negative (data points located in the upper left quadrant; SI_1_ = −0.48 ± 0.47). This means that the responses to the lower-intensity stimulus were smaller when the stimulus was presented as deviant than as standard. At the same time, SI_2_ was mostly positive (SI_2_ = 0.45 ± 0.29), indicating that the responses to the higher-intensity stimulus were generally larger when the stimulus was presented as deviant than as standard. There were 4 neurons (3.4%) with SI_1_ values larger than +0.18 (the cutoff criterion for strong SSA; see above). However, a bootstrap test (with 1000 within-neuron resamples of trials) showed that the positive SI_1_ values in these neurons arose, because the lower-intensity stimulus elicited little or no response either as standard or as deviant. When neither the standard nor the deviant elicit a significant response, the SI becomes singular and thus meaningless. Forty-four neurons (37.8%) showed an SI_1_ value of −1, indicating that the lower-intensity stimulus elicited zero response when presented as deviant.

Figure 2D shows the SI_1_ values separately for the groups of neurons with strong and weak frequency SSA. The figure shows that neurons with strong frequency SSA (red) generally also showed strong SSA in the double oddball condition, but not SSA for the intensity oddball condition (indicated by negative SI_1_ values). In contrast, the group with weak frequency SSA (blue) showed SI_1_ values close to zero in all three conditions.

### Responses to higher-intensity standards adapt responses to lower-intensity deviants

**Figure 3:**
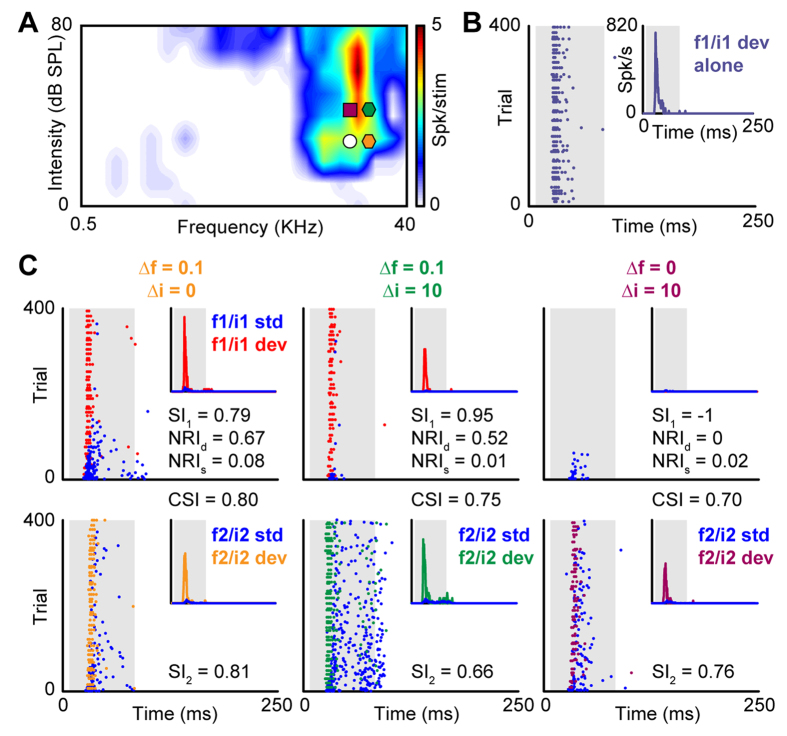
Single-neuron example of frequency, intensity and double oddball responses and corresponding SSA indices. (**A**) FRA of the neuron together with stimuli, plotted as in Fig. 1A. (**B**) Dot raster plot showing the neuron’s responses to the low-frequency/intensity sound (f1/i1; white circle in (**A**)) in the deviant-alone protocol. The inset shows the corresponding PSTH. The gray-shaded background indicates the timing of the stimulus. (**C**) Dot raster plots showing the neuron’s responses to the low-frequency/intensity sound (top row) when presented as standard (blue) or as deviant (red; see legend in leftmost panel) in the frequency oddball paradigm (left panel; orange hexagon in (**A**), the double oddball paradigm with Δ*f* = 0.1 and Δ*i* = 10 dB (right panel; green hexagon in (**A**) and the intensity oddball paradigm with Δ*i* = 10 dB (right panel; burgundy square in (**A**). As reference, in the bottom row we show the responses to the high-frequency/intensity sounds (f2/i2) when presented as standard (blue) or as deviant (orange, green or burgundy). All PSTHs shown in this figure have been normalized to the f1/i1 deviant-alone response and are thus shown in relative units. The relevant SI_1_, NRI_d_ and NRI_s_ values are also shown in each panel in the top row. The CSI values, calculated by combining the data from corresponding panels in the top and bottom row are shown between the panels. As reference, SI_2_ value is shown in the bottom row.

Whilst the CSI works well for frequency oddball paradigms, where the two stimuli are chosen to elicit similar firing rates^21^, it fails for intensity oddball paradigms, where the firing rates elicited by the two stimuli can be very different. Figure 3 shows a typical example illustrating this effect. In the frequency and double oddball conditions (Fig. 3C, left and middle panels), both the CSI and SI values correctly indicate the presence of SSA. However, in the intensity oddball condition, the CSI wrongly suggests SSA, because it is being biased by the much larger responses to the higher-intensity sound than to the lower-intensity sound (compare right panels in Fig. 3C), which means that the (positive) difference between the higher-intensity deviants and standard responses is often larger than the (negative) difference between the lower-intensity deviants and standard responses.

### Tradeoff between frequency separation and intensity difference in double oddball paradigms

**Figure 4:**
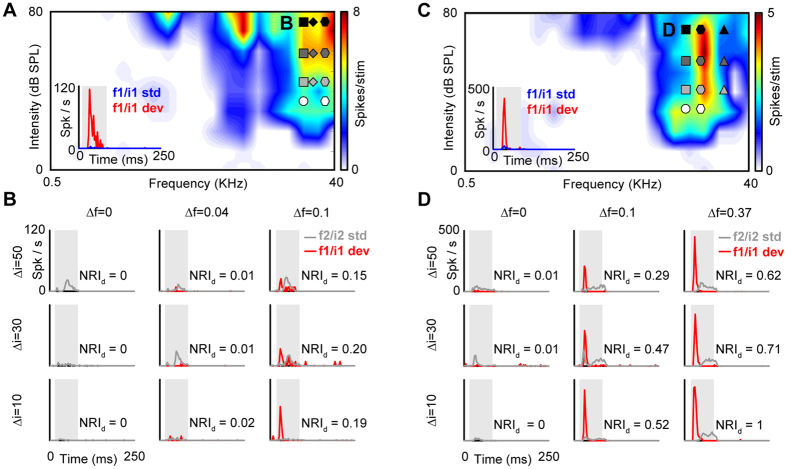
Two example neuron responses to the intensity and double oddball paradigms. (**A**,**C**) FRAs of the two neurons with intensity and double oddball stimuli, plotted as in Fig. 1A. The insets show the PSTHs for the low-frequency/intensity sound (f1/i1; white circle) presented as standard (blue) or deviant (red) in the frequency oddball condition (Δ*f* = 0.1; Δ*i* = 0; see white diamond). (**B**) PSTHs showing the responses of examples neuron #1 (panel **A**) to the low-frequency/intensity deviant sound (f1/i1 dev; red line) in nine different intensity and double oddball paradigms, with frequency separations of Δ*f* = 0, 0.04 and 0.1 (rows) and intensity differences of Δ*i* = 10, 30 and 50 dB (columns). For comparison, the gray lines show the corresponding high-frequency/intensity standard responses (f2/i2 std). (**D**) PSTHs showing the responses of examples neuron #2 (panel **C**) to the low-frequency/intensity deviant in nine different intensity and double oddball paradigms. In this case, the frequency separations were Δ*f* = 0, 0.1 and 0.37.

CSI and SI values represent the difference between the deviant and standard responses relative to the summed response. Both indices become singular and thus fail when the summed response is small (as is often the case in intensity oddball paradigms). Here, we devise a new index, referred to as the *normalized response index* (NRI) to evaluate the degree of adaptation of the deviant and standard responses separately (see Methods). We use the NRI to explore how the degree of adaptation of the deviant response depended on the frequency and intensity separation between the deviants and standards in the intensity and double oddball paradigms. Figure 4 shows deviant responses from two example neurons with strong frequency SSA. In both cases, the response to the lower-intensity deviant was practically completely suppressed (NRI_d_ ≈ 0) when the standard was at the same frequency as, and a higher intensity than, the deviant (Fig. 4B,D, left columns). A similar pattern was also observed for the smallest non-zero standard-deviant frequency separation (Δ*f* = 0.04; Fig. 4B, middle column), but for the larger frequency separations (Δ*f* = 0.1 or 0.37; Fig. 4B,D, right columns), the lower-intensity deviant started to elicit a noticeable response (NRI_d_ > 0), particularly at the smallest intensity difference (Δ*i* = 10 dB). As the intensity difference increased (Δ*i* = 30 or 50 dB), the deviant response (and thus NRI_d_) tended to decrease.

**Figure 5:**
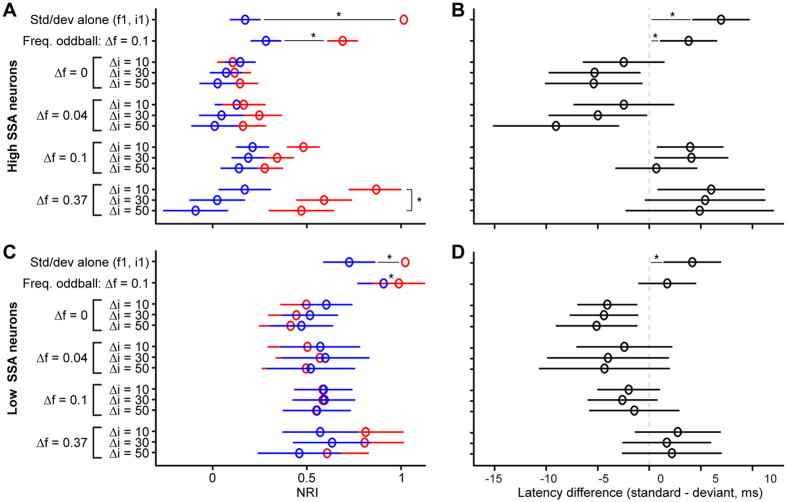
Opposing effects of frequency separation and intensity difference in double oddball paradigms. (**A**,**C**) Normalized response indices (NRIs) for the deviant (NRI_d_: red) and standard (NRI_s_: blue), averaged across neurons with strong (**A**) and weak (**C**) frequency SSA. Different oddball conditions are shown in different rows (see ordinate). The first row shows the deviant- and standard-alone conditions and the second row, the frequency oddball condition (Δ*f* = 0.1, Δ*i* = 10 dB). The error bars show the 95% confidence intervals of the means. Asterisks (*) show statistical differences. (**B**,**D**) First-spike latency differences between standard and deviant responses (std–dev: positive values mean that the deviant response has a shorter latency) for the same conditions and neurons as the NRI values shown in panels (**A**,**C)**.

In order to explore these effects further, we analyzed the average NRI values for the groups of neurons with strong and weak frequency SSA (Fig. 5A,C) and measured the average first-spike latency differences between the standard and deviant responses (for the lower-frequency/intensity sound, like the NRI values; Fig. 5B,D). In the frequency oddball condition (Δ*f* = 0.1, Δ*i* = 0; 2^nd^ row in Fig. 5A,C), both groups of neurons showed stronger adaptation of the standards than deviants (sign test of NRIs: strong-SSA, *p* < 0.001; weak-SSA, *p* = 0.011), but the difference was much larger for the strong- than weak-SSA neurons (Wilcoxon rank sum test: *p* < 0.001). This was expected given the way the weak- and strong-SSA neurons were defined. Both neuron groups also showed stronger adaptation in the standard-alone than deviant-alone condition (1^st^ rows in Fig. 5A,C; sign test of NRIs: both *p* < 0.001), but, again, the difference was much smaller for the weak-SSA than strong-SSA neurons (Wilcoxon rank sum test: *p* < 0.001). This indicates that, in weak-SSA neurons, adaptation is not only less specific, but the level of adaptation is also overall much weaker. This will be further explored in the modelling section below. The NRI values for the intensity and double oddball conditions were compared with linear mixed-effects models, with Δ*f*, Δ*i* and stimulus type (standard, deviant) as fixed factors and neuron as random intercept. The analysis of the strong-SSA neurons yielded significant main effects of all factors [Δ*f*, Δ*i*, stimulus type; χ^2^(3) = 110.07, χ^2^(2) = 30.89, χ^2^(1) = 80.27, respectively; all *p* < 0.001], significant two-way interactions [Δ*f* × Δ*i*: χ^2^(6) = 21.21; Δ*f* × stim: χ^2^(3) = 132.56; Δ*i* × stim: χ^2^(2) = 7.36; *p* ≤ 0.025] and a significant three-way interaction [χ^2^(6) = 19.58, *p* = 0.003]. The two-way interaction between Δ*f* and stimulus type shows that the difference between the standard and deviant NRIs increased with increasing standard-deviant frequency separation. The three-way interaction was caused by a significant Δ*i* by stimulus type interaction for Δ*f* = 0.37 [χ^2^(2) = 18.72, *p* < 0.001], a marginal interaction for Δ*f* = 0.1 [χ^2^(2) = 5.39, *p* = 0.068], but non-significant interactions for Δ*f* = 0.04 and 0 [χ^2^(2) = 3.73, *p* = 0.155]. These results confirm that, in the strong-SSA neurons, there was a tradeoff between frequency separation and intensity difference, with greater deviant responses for larger frequency separation, but smaller responses for greater intensity differences. In contrast to the strong-SSA neurons, the weak-SSA neurons showed significant main effects of Δ*f* and Δ*i* [χ^2^(3) = 23.75, χ^2^(2) = 10.71; *p* ≤ 0.005], but not of stimulus type [χ^2^(1) < 0.01, *p* = 0.957]. In addition to the main effects, only the Δ*f* by stimulus type interaction was significant [χ^2^(3) = 29.99; *p < *0.001]. The interaction arose because of significant main effects of stimulus type for Δ*f* = 0 and 0.37 [χ^2^(1) = 16.30, χ^2^(1) = 11.66, respectively; both *p* < 0.001], but not for Δ*f* = 0.04 and 0.1 [χ^2^(1) = 0.40, χ^2^(1) = 0.78, respectively; both *p* ≥ 0.38]. For Δ*f* = 0, NRI_d_ was generally smaller than NRI_s_, whereas, for Δ*f* = 0.37, NRI_d_ was larger than NRI_s_. This shows that, even for the weak-SSA neurons, the standard-deviant frequency separation had a small, but noticeable effect on the standard and deviant responses.

The first-spike latency differences (Fig. 5B,D) were consistent with the NRI values, in that conditions where NRI_d_ was larger than NRI_s_ (the deviant response was less adapted than the standard response) tended to yield positive latency differences (shorter deviant than standard latencies), whereas conditions where NRI_d_ was smaller than NRI_s_ tended to yield negative latency differences (longer deviant than standard latencies). In the strong-SSA neurons, the largest positive latency differences were observed between the standard- and deviant alone conditions and for the frequency oddball condition (1^st^ and 2^nd^ rows in Fig. 5B; Wilcoxon rank sum: both p < 0.001). Positive latency differences were also observed for the larger frequency separations (Δ*f* = 0.1 and 0.37) in the double oddball conditions, particularly at the smaller intensity differences, whilst negative latency differences were observed for the intensity oddball condition (Δ*f* = 0) and the double oddball condition with the smallest frequency separation (Δ*f* = 0.04). The weak-SSA neurons showed a similar pattern of results, but with latency differences that were generally closer to zero than for the strong-SSA neurons. Statistical analyses using linear mixed effects models with Δ*f* and Δ*i* as fixed factors and neuron as random intercept revealed a significant main effect of Δ*f* for both the strong- [χ^2^(3) = 40.92; *p* < 0.001] and weak-SSA neurons [χ^2^(3) = 22.07; *p* < 0.001], and a marginal main effect of Δ*i* for the strong-SSA neurons [χ^2^(2) = 5.41; *p* = 0.067], but not for the weak-SSA neurons [χ^2^(2) = 0.37; *p* = 0.831]. The Δ*f* by Δ*i* interaction was non-significant for both groups [strong-SSA: χ^2^(6) = 3.84; weak-SSA: χ^2^(6) = 0.86; both *p* ≥ 0.699].

### Absence of intensity SSA even in neurons with non-monotonic rate-intensity functions

In marmoset monkey auditory cortex, intensity-tuned neurons (*i.e.*, neurons with non-monotonic rate-intensity functions) can adapt to frequent loud sounds whilst at the same time maintaining their sensitivity to rare fainter sounds^28^,^29^. Here, we tested whether the same was true for IC neurons with non-monotonic rate-intensity functions. For that, we first classified the neurons using the monotonicity index^35^ (MI; see Methods), dividing them into monotonic (MI ≥ 0.75) and non-monotonic groups (MI < 0.75). If the non-monotonic neurons maintained responsiveness to the lower-intensity deviants, the lower-intensity deviant response should be less suppressed compared to the unadapted response, and so, NRI_d_ for intensity and double oddball conditions should be larger in the non-monotonic, compared to the monotonic, neurons. We compared the NRI_d_ values for all intensity and double oddball conditions tested (all intensity differences and frequency separations), but found no significant differences in NRI_d_ between the monotonic and the non-monotonic neurons in any condition (Mann-Whitney rank sum test with a Holm-Bonferroni correction, *p* > 0.1 in all cases).

### Neurons with strong frequency SSA have narrowly-tuned adaptation channels

**Figure 6:**
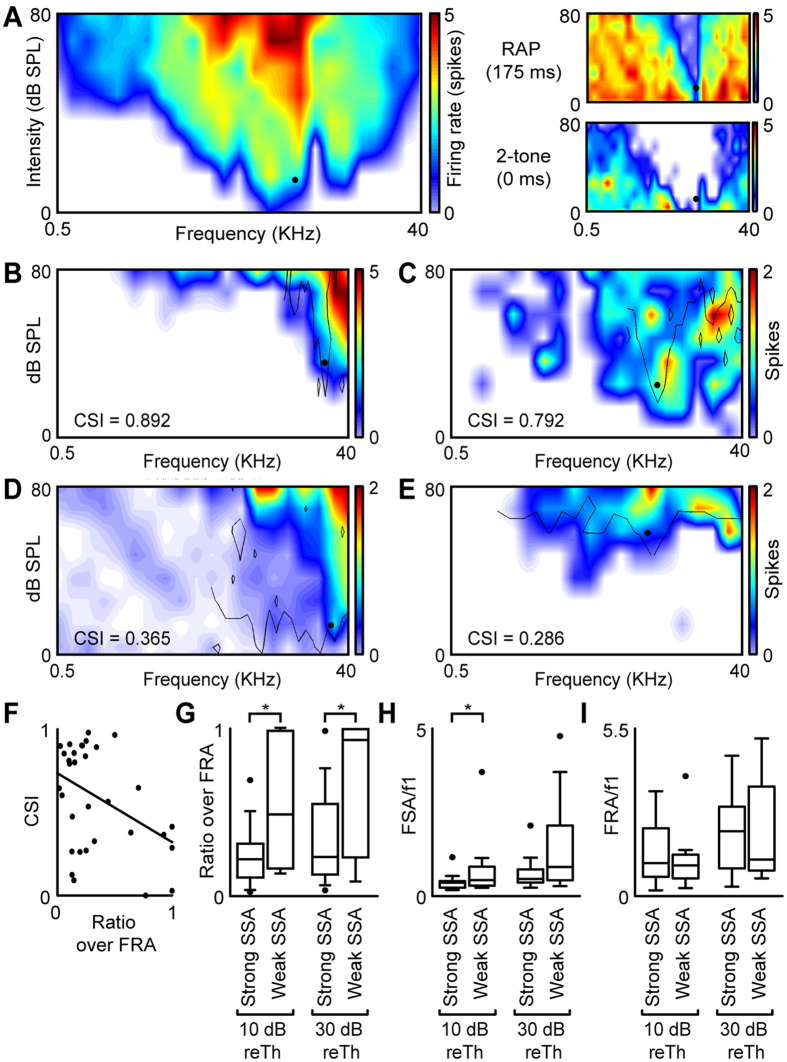
Properties of adaptation channels as measured by the repeated adaptation paradigm (RAP). (**A**) Left panel: FRA of an example IC neuron showing the probe sound used in the RAP as a black dot. For the same neuron, the upper right panel shows the probe responses in the RAP, and the bottom right panel in a forward-suppression paradigm. (**B**,**C**) FRAs of two neurons with strong frequency SSA and corresponding FSAs (black lines). (**D**,**E**) FRAs of two neurons with weak frequency SSA and corresponding FSAs. (**F**) Relationship between CSI and the FSA-to-FRA ratio at 10 dB above the probe level. (**G**) FSA-to-FRA ratio in the neurons with strong and weak frequency SSA at 10 and 30 dB above the probe level. (**H**,**I**) FSA (**H**) and FRA (**I**) width relative to the probe frequency, again evaluated for neurons with strong and weak frequency SSA and at 10 and 30 dB.

The results so far are consistent with the idea that frequency SSA in the IC is caused by independent adaptation within convergent frequency-tuned input channels (adaptation channels) to the IC neurons. Here, we developed a *repeated adaptation paradigm* (RAP) to measure the widths of the adaptation channels in relation to the neurons’ overall tuning widths (reflected by the FRA). The RAP measures the suppression of the response to a “probe” stimulus when the probe is preceded by an “adaptor” stimulus, presented repeatedly to mimic the conditions of the oddball paradigm (see Methods and Fig. 1C). Figure 6A shows the FRA of an example neuron (large panel on the left) together with the area of frequencies and intensities within which the adaptor caused suppression of the probe response (referred to as frequency suppression area, or FSA; upper right panel). The FSA obtained with the RAP was much narrower than that obtained with a forward-suppression paradigm^36^,^37^ (lower right panel). In forward-suppression paradigms, the adaptor is presented only once and the gap between the adaptor and probe is usually short. We tested such a forward-suppression protocol (with a 0-ms gap between the adaptor and probe) in 7 of the 33 neurons in which we tested the RAP. The suppression area obtained with the forward-suppression protocol typically covered the whole of the FRA. This is consistent with the findings by Scholes *et al.*^37^, who reported forward-suppression areas that were typically as wide as the FRA.

Of the 33 neurons tested with the ROP, neurons with strong frequency SSA tended to show FSAs that were narrow in relation to their FRAs (see Fig. 6B,C for two examples), whereas neurons with weak frequency SSA tended to show FSAs that were broader (see Fig. 6D,E). For the neurons with strong SSA (CSI ≥+0.18; see above), of which there were 21, the FSA covered barely a quarter of the FRA, on average, at both 10 and 30 dB above the probe intensity. In contrast, for the neurons with weak SSA (*n* = 12), the FSA covered around half of the FRA at 10 dB and almost all the FRA at 30 dB (Fig. 6F). A Wilcoxon rank sum test showed that this difference was significant (*p* = 0.0247 and 0.0312, respectively; Fig. 6G). The narrower relative width of the FSA compared to the FRA for the strong-SSA neurons could be because, either the suppression area was narrower, or the FRA was wider in strong- than weak-SSA neurons. To test which, we also calculated the widths of the FSA and FRA separately relative to the best frequency of the neuron (inverse of the respective Q-values). The relative width of the FSA was significantly smaller in the strong- than weak-SSA neurons at 10 dB above probe intensity (Wilcoxon rank sum test, *p* = 0.0494), and marginally smaller at 30 dB (*p* = 0.0793; Fig. 6H). In contrast, the relative width of the FRA was not significantly different between strong- and weak-SSA neurons (at either level; Wilcoxon rank sum tests: all *p* ≥ 0.443; Fig. 6I). This suggests that strong-SSA neurons have narrower adaptation channels than weak-SSA neurons.

### The width of adaptation channels is frequency-dependent

**Figure 7:**
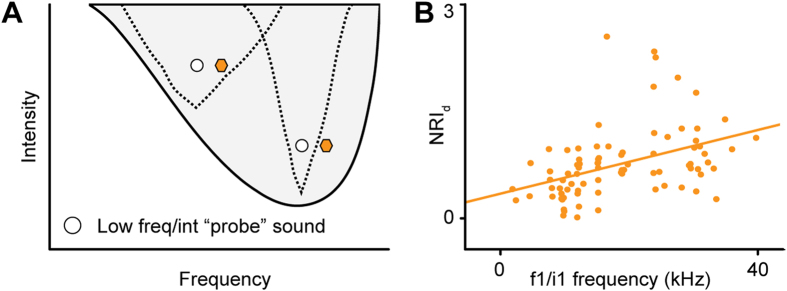
Adaptation specificity decreases with increasing frequency. (**A**) Schematic FRA showing the adaptation channels (dashed lines) for two frequency oddball conditions (white and colored symbols), one where the low-frequency/intensity sound (white circle) is set at a low frequency (left) and where it is set at a higher frequency (right). (**B)** Relationship between the NIA_d_ values in the frequency oddball paradigm (Δ*f* = 0.1, Δ*i* = 10 dB) and the frequency of the low-frequency/intensity sound (f1/i1 frequency). The solid line shows the regression line.

Recent results by Duque and colleagues^32^ have shown that SSA tends to be stronger at low intensities and at the high-frequency edge of the FRA. The results from the previous section suggest that strong frequency SSA is associated with narrower adaptation channels. In light of this finding, the results by Duque and colleagues suggests that (i) adaptation channels are narrower when the sound intensity is low, and (ii) adaptation channels tuned to higher frequencies are also narrower (Fig. 7A). To test whether the current data support this hypothesis, we analyzed the dependence of NRI_d_ on the frequency (*f*_*1*_) and intensity (*i*_*1*_) of the deviant stimulus. We applied a linear regression model and ANOVA, with *f*_*1*_ and *i*_*1*_ as fixed covariates, to the data from the frequency oddball paradigm (Δ*f* = 0.1, Δ*i* = 0). As in the study by Duque and colleagues^32^, the analysis was limited to neurons with strong frequency SSA (CSI ≥ +0.18; see above). It revealed a significant main effect of *f*_*1*_ on NRI_d_ [*F*(1,116) = 14.21, *p* < 0.001], with greater NRI_d_ values being associated with higher *f*_*1*_ values (Fig. 7B; Pearson’s r^2^ = 0.172, *p* < 0.001). This confirms that the relative width of the adaptation channels decreases with increasing frequency. However, we didn’t find any effect of deviant intensity.

### Adaptation channel model

**Figure 8:**
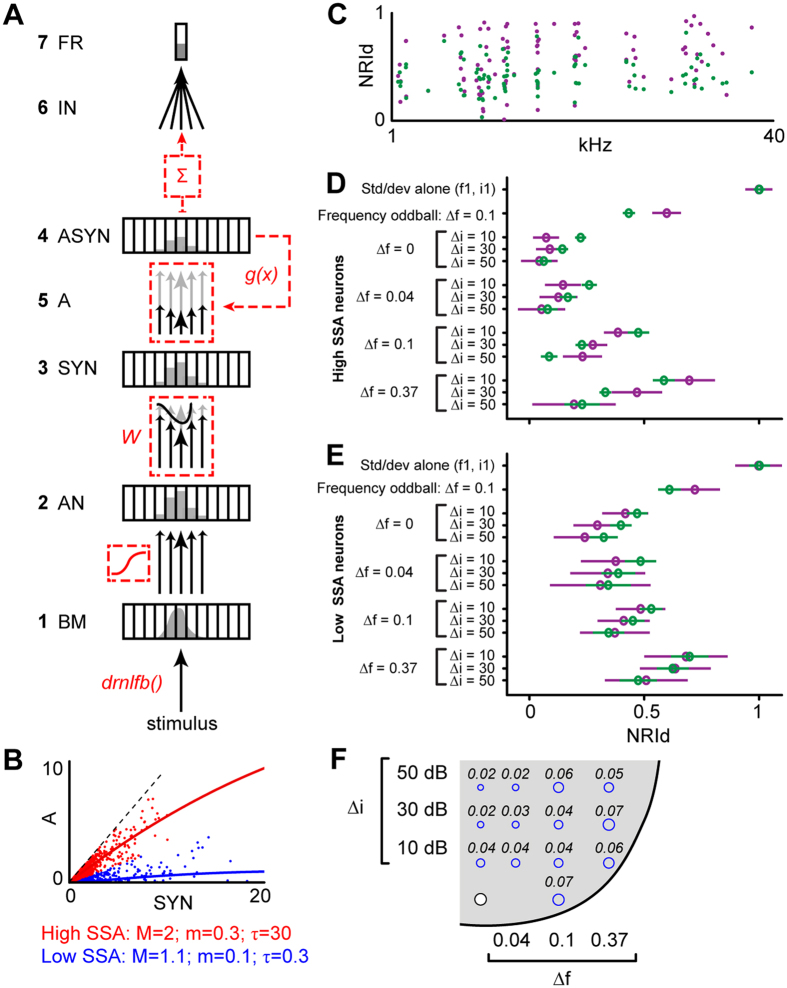
Comparison between adaptation model results and data. (**A**) Summary of the computational model of synaptic adaptation used to simulate the measured oddball responses. (**B**) Relationship between adaptation (estimated by difference in firing rate between standard-alone and deviant-alone conditions) and synaptic activity (estimated from firing rate for deviant-alone condition), shown separately for low- (blue) and strong-SSA neurons (red). (**C**) Comparison of experimental (burgundy) and simulated (green) NIA_d_ values of individual neurons in the frequency oddball condition (*∆f* = 0.1). (**D**,**E**) Comparison of average experimental (burgundy) and simulated (green) NRI_d_ values for all oddball conditions, plotted as in Fig. 5. As in Fig. 5, the NRI_d_ values were averaged across the strong- (**D**) and weak-SSA (**E**) neurons separately. (**F**) Mean squared deviation between experimental and simulated NIA_d_ for each oddball condition. The size of the deviation is indicated by the size of the blue circles. The white circle shows the position of the lower-frequency-intensity stimulus with the stimulus space.

Here, we test whether the current data are consistent with a simple model based on the assumption that SSA in the IC arises as a result of adaptation that occurs independently within the auditory frequency channels formed in the cochlea. Adaptation was assumed to arise as a result of activity-dependent synaptic depression. Each adapting synapse was assumed to be activated by a single cochlear frequency channel (Fig. 8A, for details, see Methods). The tuning of the cochlear channels was modeled with a non-linear filterbank model^38^. The amount of adaptation within each synapse was assumed to increase with increasing activation of that synapse and recover over time, and was subtracted from the new activation by the next stimulus. The new activation causes new adaptation, which was added to the remaining adaptation from previous trials. Adaptation was assumed to be generally proportional to activation, but in order to accommodate the possibility that adaptation properties differ between low- and high-threshold auditory nerve fibers, the proportionality constant (see Methods) was allowed to differ between low (*M*) and high (*m*) levels of synaptic activity. Figure 8B shows population values of *M* and *m*, fitted to all weak- (blue) or strong- (red) SSA neurons. In line with the results from the NRI values (see above), these population fits suggest that the same amount of synaptic activity (estimated by the deviant-alone responses) caused much lower levels of adaptation (estimated by the difference between the standard- and deviant-alone responses) in the weak- than strong-SSA neurons. Figure 8C shows the simulated individual NRI_d_ values for one example condition (frequency oddball, *∆f* = 0.1). The simulated data points (green dots) cover generally the same range as the measured data points (burgundy dots). Figure 8D,E shows the simulated average NRI_d_ values for all measured conditions, plotted separately for weak- and strong-SSA neurons as in Fig. 5A,C. In line with the data, the model showed no SSA for intensity oddball conditions, and for double oddball conditions with small frequency separations or large intensity differences. Moreover, the model also replicated the differences in the pattern of NRI_d_ values between the strong- and weak-SSA neurons (compare Fig. 8D,E). Overall, the model explained about 60% of the variability in the data, with similar accuracy across all conditions (Fig. 8F).

## Discussion

The current results show that, while neurons in the IC exhibit strong SSA to frequency deviants, they fail to show SSA to pure intensity deviants. This was true even for neurons with non-monotonic rate-intensity functions. The response to low-intensity deviants among high-intensity standards was strongly suppressed or abolished unless the deviants and standards also differed in frequency. This suggests that, in the IC, SSA arises as a result of independent adaptation within the IC’s frequency-tuned input channels. The current results show that the relative width of these adaptation channels decreases with increasing frequency and that neurons with strong frequency SSA integrate over multiple such channels.

Two previous studies measured responses to intensity oddball paradigms in the mammalian auditory cortex^17^,^26^. Like the current study, both found enhanced responses to high- but not low-intensity deviants. Despite finding similar results, one study concluded the presence of intensity SSA^17^, whereas the other concluded the opposite^26^. Interpretations as to whether or not SSA is present can depend on the metric by which SSA is measured. The current study used the normalized response index (NRI), which measures the degree of adaptation of the standard and deviant responses separately and is particularly suited for conditions where the standards and deviants elicit different-sized responses. The current NRI results are consistent with the interpretation by Farley and colleagues^26^ and suggest the absence of intensity SSA also in the IC. However, results by Reches and Gutfreund^39^ indicate that neurons in optic tectum gaze control system in the barn owl do show SSA to low-intensity deviants, suggesting that they exhibit deviance sensitivity similar to the MMN. This is consistent with their functional role in exogenous attentional orienting^40^.

An interesting aspect of the current results was that, whilst most of the neurons with strong frequency SSA showed a complete, or close to complete, suppression of the low-intensity deviant response in the intensity oddball paradigm, neurons with weak frequency SSA showed low-intensity deviant responses that were often similar in size to the low-intensity standard responses. The NRI values suggest that this was, because weak-SSA neurons were generally much less adapted than strong-SSA neurons (i.e., the same amount of synaptic activity caused much less adaptation in weak- than strong-SSA neurons). A speculative interpretation of this finding is that the IC contains a subset of neurons specialized in frequency-specific adaptation. The current data suggest that there is no corresponding subset of IC neurons specialized in intensity-specific adaptation, because even neurons with non-monotonic rate-intensity functions failed to show intensity SSA. This finding is consistent with the idea that non-monotonicity arises at, rather than before, the level of the IC, for instance through intra-collicular inhibitory projections^65^,^66^. Previous results suggest that inhibition within the IC is not involved in the generation of SSA^53^. In contrast to the IC, it has been shown that some non-monotonic neurons in auditory cortex adapt specifically to high-intensity sounds, whilst preserving their sensitivity to low-intensity sounds^28^,^29^. Such neurons might receive convergent input from lower-level non-monotonic neurons with different intensity response ranges.

Previous modeling work has shown that gain adaptation within a single layer of frequency-tuned neurons is unable to explain all aspects of SSA^30^,^41^, and that more complicated networks of adapting neurons are needed to explain some of the effects observed in previous data^42^,^43^. However, the current results are largely consistent with the idea that SSA in the IC arises as a result of independent adaptation within narrowly frequency-tuned channels. The results from the RAP showed that neurons with strong frequency SSA integrate over multiple narrowly frequency-tuned channels, whereas neurons with weak frequency SSA receive input from fewer and wider channels. The data from the oddball paradigms were broadly consistent with a simple gain adaptation model based on the assumption that adaptation occurs independently within the auditory frequency channels. The model replicated the finding that responses to low-intensity deviants were adapted by high-intensity standards, because frequency-tuned channels responsive to a low-intensity sound will also respond to, and thus be adapted by, a high-intensity sound at the same frequency. The model was also consistent with the data from the double oddball conditions: as the frequency separation between the standard and deviant increased, the standard and deviant activated more disparate frequency channels, and so, the deviant response became less adapted; as the intensity difference increased, the standard activated a greater range of channels, including those responsive to the deviant, and so, the deviant response became more adapted. However, gain adaptation within a single layer of frequency-tuned neurons cannot explain the current finding that the adaptation channels measured with the RAP were narrower than those measured with a forward-suppression paradigm. Forward suppression refers to the reduction in the response to a probe stimulus when preceded by a single adaptor^36^,^37^. Comparison between the RAP and forward suppression results suggest that the degree of adaptation specificity increases with repeated exposure to the adapting sound. The current forward suppression paradigm used a shorter gap between the adaptor and probe than the RAP. However, a recent electroencephalographic study has demonstrated a higher degree of adaptation specificity with repeated than with single adaptors, even when all stimuli were separated by the same, long gap^41^. These findings, suggest that adaptation mechanisms change with repeated exposure to the adapting sound. Mill and colleagues^42^ have shown that increased adaptation specificity for repeated adaptors can be explained with a two-layered adaptation model. Mill *et al.*’s model consists of a first layer of sharply-tuned converging synapses, similar to the adapting synapses in the current model, but then contains another layer of synapses, which are more broadly tuned as a result of the convergence in the first layer. The second layer enables the model to explain the change in adaptation specificity between single and repeated adaptors. In the RAP, each probe was preceded by only three adaptors. In the current oddball paradigms, the number of standards preceding each deviant was typically much larger (9:1 ratio). This suggests that adaptation channels were even narrower during the oddball paradigm than measured with the RAP.

The double oddball conditions never yielded stronger SSA than the frequency oddball condition. This was, because IC neurons showed little or no intensity SSA. Previous studies have shown that the MMN is also no larger for double deviants than for pure frequency deviants^44^,^45^,^46^. However, in contrast to SSA in the IC, the MMN can be elicited by pure intensity deviants, and so, the finding that the MMN to double deviants is no larger than to frequency deviants indicates that the MMN is non-additive, and thus, that it processes information from different deviant types (frequency and intensity) together rather than independently (see, however, Althen *et al.*^47^).

Using intensity oddball sequences similar to the current ones, Jacobsen and colleagues^10^ found that lower-intensity deviants elicited an MMN, but no enlarged N1 response. The N1 is thought to reflect stimulus-driven activity within non-primary auditory cortical areas^63^,^64^, and so, the finding that the N1 is insensitive to intensity deviants would suggest that MMN-like deviance sensitivity first emerges at higher levels of non-primary auditory cortical processing. However, Althen and colleagues^27^ measured electroencephalographic middle-latency responses (MLRs) to intensity oddball sequences and found a slight shift in the transition between the Na and Pa peaks in response to low-intensity deviants versus standards. This shift caused a small negative deflection in the difference wave between the deviant and standard responses, which Althen and colleagues interpreted as an early component of the intensity-change MMN. Given that the MLR is thought to be generated in between the thalamus and the primary auditory cortex^65^,^66^,^67^, one might suggest that MMN-like deviance sensitivity first emerges at or before the level of primary auditory cortical processing. However, rather than representing an early MMN component, the observed shift in the transition between the Na and Pa may also have been caused by a suppression in the amplitude, and/or a prolongation in the latency, of the Pa response to the low-intensity deviant. The latter suggest that intensity oddball effects in the MLR are similar to those observed here in IC single-neuron responses (responses to low-intensity deviants were suppressed had longer first-spike latencies). This would be consistent with the N1 results by Jacobson and colleagues suggesting that MMN-like deviance sensitivity emerges only at later stages of non-primary auditory cortical processing.

The oddball paradigm represents a special case of a random stimulus distribution, in which the stimulus can take one of only two discrete values (standard and deviant). A wide range of previous studies on neural adaptation have used continuous, rather than discrete, stimulus distributions, where the stimulus can take one of a whole range of different values^2^. These studies have shown that, when the stimulus is varied along a dimension that elicits a monotonic stimulus-response function, the response range tends to adjust to both the mean and variance of the stimulus distribution in a way that enhances the encoding of the most commonly occurring sounds (referred to as dynamic-range adaptation^3^,^48^,^49^). Dean and colleagues^48^ presented sounds at a wide range of intensities, all with the same, low probability of occurrence, apart from a narrow high-probability range. They found that the response range of neurons in the IC shifted towards the high-probability intensities. As a result, the responses to low-probability low intensities were all but completely suppressed when the high-probability intensities were high, but when the high-probability intensities were low, both low and high intensities produced larger responses. This pattern of result is similar to the current pattern of results for the intensity oddball paradigm, potentially suggesting common underlying mechanisms. However, dynamic range adaptation involves processes with time-scales that would appear to be too fast to affect responses to oddball paradigm stimuli^2^,^50^,^51^. Thus, some of the processes that contribute to dynamic-range adaptation may be shorter-lived than the processes underlying the adaptation effects observed in the current study. Instead, they may be more akin to the processes underlying forward suppression^36^,^37^.

In summary, our results demonstrate a lack of SSA for intensity deviant sounds in the IC, although SSA occurred for double-deviants when standard and deviant activate sufficiently disparate frequency channels. Moreover, the current results indicate that gain adaptation within frequency-tuned input channels is an important component of SSA at the level of the midbrain.

## Methods

### Surgical procedures

Experiments were performed on 37 adult pigmented female rats (*Rattus norvergicus*, Long-Evans) with body weights between 150 and 260 g. All experimental procedures were carried out at the University of Salamanca using methods conforming to the standards of, and approved by, the University of Salamanca Animal Care Committee. Urethane was used to induce (1.5 g/kg, i.p., 20% solution) and maintain (0.5 g/kg, i.p. given as needed) anesthesia. Urethane was chosen as an anesthetic because its effects on multiple aspects of neural activity (including inhibition, spontaneous firing and SSA^52^,^53^) are known to be less than those of barbiturates and other anesthetic drugs^54^ (generally specific to inhibitory receptors). Details of surgical procedures have been described previously^19^,^55^. In brief, the animal was placed in a stereotaxic frame, located inside a sound-attenuated room and with the ear bars replaced by hollow specula to accommodate the sound delivery system. The scalp was incised along the sagittal midline, and the skin reflected laterally before performing a craniotomy to expose the cerebral cortex overlaying the left IC.

### Electrophysiological recording

Extracellular single-unit responses were recorded using a tungsten electrode (1–2 MΩ^56^,^68^) lowered through the cortex by means of a piezoelectric microdrive (Burleigh 6000 ULN). Electrode positioning was based on stereotaxic coordinates, physiological criteria (tonotopicity and other response properties^57^,^58^) and confirmed histologically after experiment termination. The sound stimuli were presented monaurally to the ear contralateral to the recording side. The sound delivery system was the same as described previously^19^,^55^. Search stimuli were pure tones or noise bursts delivered using TDT System II hardware and custom software^19^,^58^,^59^. The sound system was calibrated using a ¼” condenser microphone (model 4136, Brüel & Kjær) and a dynamic signal analyzer (Photon+, Brüel & Kjær). The maximum sound system output was flat between 0.3–5 kHz (~100 ± 7 dB SPL) and between 5–40 kHz (~90 ± 5 dB SPL). The system’s frequency output was limited to 40 kHz. Even at the highest output level, the system’s relative distortion level was less than −40 dB. Details about the electrophysiological setup and procedures have been described previously^19^,^55^. The spike times were logged with a resolution of ~150 μs. For each neuron, the monaural frequency response area (FRA; *i.e.*, the combination of frequencies and intensities capable of evoking a response) was determined by presenting pure tones (75-ms duration with 5-ms rise and fall times) at a range of different frequencies (from 0.5–40 kHz, in 25 logarithmic steps, presented randomly) and intensities (in 10 dB steps, presented in order from lowest to highest) using an automated procedure. Each frequency and intensity combination was repeated 5 times.

### Stimulus presentation paradigms

The FRA was used to set the stimulus parameters for the frequency-, intensity- and double- (frequency and intensity) oddball paradigms. For the frequency oddball paradigm, we choose a pair of frequencies (*f*_*1*_ and *f*_*2*_) that, at the same intensity of *i*_*1*_ = *i*_*2*_ ≈ 10 dB above the best-frequency threshold, elicited a similar firing rate (Fig. 1A). The relative separation of the frequencies, Δ*f*, was fixed at 0.10 (corresponding to 0.141 octaves), where Δ*f* = (*f*_*2*_ − *f*_*1*_)/(*f*_*2*_ · *f*_*1*_)^1/2^. The two frequencies were presented in a random (“oddball”) sequence consisting of 400 stimuli in total, with the lower frequency (*f*_*1*_) presented in 90% of the trials (“standard”) and the higher frequency (*f*_*2*_) presented in 10% of the trials (“deviant”). The stimulus onset asynchrony (SOA) was 250 ms (corresponding to a 4 Hz stimulus presentation rate). After completion of the first sequence, a second sequence was presented, in which the roles of the two stimuli were reversed (*f*_*2*_ was standard and *f*_*1*_ was deviant). Similar oddball sequences have previously been shown to evoke strong SSA in IC neurons^19^,^32^. In addition to the two oddball sequences, we also measured the response to the lower-frequency stimulus (*f*_*1*_) in a deviant-alone and a standard-alone sequence, which were similar to the oddball sequences, but with the standards or deviants replaced by silence, respectively. We assumed that the response in the deviant-alone condition would be largely unadapted, and thus represent the maximum-possible response of the neuron at the relevant frequency and intensity (*f*_*1*_, *i*_*1*_).

For the intensity and double oddball conditions, we fixed the frequency and intensity of the lower-frequency stimulus (*f*_*1*_, *i*_*1*_) and varied the frequency and/or intensity of the higher-frequency stimulus (*f*_*2*_, *i*_*2*_; Fig. 1A), where *i*_*2*_ was always greater than *i*_*1*,_ and *f*_*2*_ was greater than or equal to *f*_*1*_. The intensity difference (Δ*i* = *i*_*2*_ − *i*_*1*_) was small (10 dB), medium (20–30 dB) or large (40–50 dB). The frequency separation (Δ*f*) was 0, 0.04, 0.1 or 0.37 (corresponding to 0, 0.057, 0.141 or 0.526 octaves, respectively). As before, two oddball sequences were presented for each condition, one in which the lower-frequency/intensity stimulus (*f*_*1*_, *i*_*1*_) was the deviant and the higher-frequency/intensity stimulus (*f*_*2*_, *i*_*2*_) was the standard, and another in which the roles of the stimuli were reversed. Fixing *f*_*1*_ and *i*_*1*_enabled us to systematically map the effect of a higher-intensity standard on a lower-intensity deviant. Figure 1B shows three example conditions, including the frequency oddball paradigm (Δ*f* = 0.1, Δ*i* = 0 dB; orange hexagon), one of the intensity oddball paradigms (Δ*i* = 10 dB, Δ*f* = 0, burgundy square) and one of the double oddball paradigms (Δ*f* = 0.1, Δ*i* = 10 dB; green hexagon). Data were collected first for the smallest intensity difference (Δ*i* = 10 dB), using at least two different frequency separations. Then, the rest of the intensity differences were measured in ascending order, using the same frequency separations as before. The complete protocol comprised a total of 14 conditions (1 frequency oddball condition +3 intensity differences × 4 frequency separations +1 deviant- and standard-alone condition; Fig. 1A) and took ∼80 min to measure in one neuron. The decision to limit *f*_*2*_ to be greater than or equal to *f*_*1*_ was necessary to achieve a manageable measurement time and was based on the finding that SSA tends to be stronger within the high-frequency part of the neuron’s FRA^32^.

In addition to the standard oddball paradigms, we also measured responses to a repeated adaptation paradigm (RAP, Fig. 1C). Like the adaptation paradigms used in previous studies^36^,^37^, the RAP is a trial-based paradigm. Within each trial, a probe tone, *p*, with a fixed frequency and intensity equal to those of the lower-frequency/intensity stimulus used in the oddball paradigms (*f*_*1*_, *i*_*1*_), was preceded by three identical adaptor tones, *c*, to create a discrete oddball sequence, *a a a p* (Fig. 1C). Within the sequences, the stimuli were presented with an SOA = 250 ms and successive sequences were separated by recovery gap of 1000 ms, making each trial 2000 ms long. The adaptors in the RAP paradigm mimic the standards in the classical oddball paradigm. The fact that the RAP paradigm uses discrete oddball sequences means that the adaptor effect can be mapped out systematically as a function of the adaptor frequency and intensity. The adaptor was presented at the same range of frequencies (0.5–40 kHz, in 25 logarithmic steps, presented randomly) and intensities (in 10 dB steps, presented in order from lowest to highest) as used for the FRA measurements. Each combination of adaptor frequency and intensity was repeated 4 times. The resulting firing rates show the area of frequencies and intensities within which the adaptor suppresses the probe response (frequency suppression areas, FSA). The bandwidth of the FSA was defined as the width of the area corresponding to a criterion level of suppression relative to a baseline, measured at an adaptor level of 10 or 30 dB SPL above the probe level. The baseline was the average probe response following the adaptors at the very lowest adaptor intensities used, where the adaptors elicited little or no discernable response. The criterion suppression was 0.6^32^.

### Data analysis

Standard and deviant responses obtained from the oddball paradigms were visualized as dot rasters and expressed in spikes per stimulus (to account for the different numbers of presentations) in a peri-stimulus time histogram (PSTH). SSA was quantified in three different ways. First, we calculated the common SSA index, CSI = [d_1_ + d_2_ − s_1_ − s_2_]/[d_1_ + d_2_ + s_1_ + s_2_], where d_*1/2*_ and s_*1/2*_ are the responses to the lower- and higher-frequency/intensity stimulus (*f*_*1/2*_, *i*_*1/2*_) when presented as deviant or standard. We also calculated the SSA index for each frequency separately, SI_1/2_ = [d_*1/2*_ − s_*1/2*_]/[d_*1/2*_ + s_*1/2*_]. Both of these types of indices have been used previously to quantify SSA for frequency oddball paradigms^17^,^19^. They can range from −1 to +1, being positive when the response to the deviant stimulus is greater. SIs and CSIs become unreliable when both the standard and deviant responses are small. Therefore, we also calculated *normalized response indices* (NRIs) of the deviant and standard responses. The responses to the lower-frequency/intensity stimulus presented as deviant or standard (d_*1*_ or s_*1*_) were normalized by the corresponding unadapted response, u_*1*_ (estimated by the deviant-alone condition), to generate the deviant or standard NRI (NRI_d_ = d_*1*_/u_*1*_, NRI_s_ = s_*1*_/u_*1*_). The NRI can range between 0 and 1, being 1 if the response to the sound is not adapted and 0 if the response to the sound is completely suppressed.

The monotonicity index^35^ (MI) was evaluated at the lower stimulus frequency (*f*_*1*_) as the firing rate at the highest intensity used in the FRA measurements, FR(*f*_*1,*_max[*i*]), divided by the highest firing rate across all lower intensities used, max[FR(*f*_*1*_*, i*)]: MI(*f*_*1*_) = FR(*f*_*1,*_max[*i*])/max[FR (*f*_*1*_, *i*)].

The figures were generated in Sigmaplot 11 (Systat Software) and Matlab (MathWorks). The statistical analyses were conducted in Sigmaplot 11, Matlab and R^60^. The NRI values were analysed with linear, or linear mixed-effects, regression models using the *lm* and *lmer* functions in R (*lmer* is part of the *lme4* package^61^). When using mixed-effects models, factors were significance-tested using likelihood ratio tests. Main effects were post-hoc tested using the testFactors function of the phia package for R^62^.

### SSA model

Neural responses to frequency, intensity and double oddball sequences were simulated using a model based on the assumption that SSA is generated by synaptic depression within narrowly frequency-tuned, convergent channels. The model consisted of seven stages (Fig. 8A). The first stage (BM) used the dual resonance nonlinear (*drnl*) filterbank^38^ to model the basilar membrane velocity within each cochlear frequency channel (*BM*_*t*_ in m/s) in response to the stimulus at time *t* (*STIM*_*t*_): *BM*_*t*_ = *drnl(STIM*_*t*_). The *drnl* filterbank models the human cochlea; therefore all stimulus frequencies were first converted from the rat to the human hearing range. The *drnl* reproduces the nonlinear changes in the shape and width of the cochlear frequency channels with frequency and intensity. The second stage (AN) computed the auditory nerve activity within each channel (*AN*_*t*_, in spikes per second) from *BM*_*t*_ using a typical threshold-saturating in/out neural function [*AN*_*t*_ = *io(BM*_*t*_)]. The third stage (SYN) modeled the synaptic activation for each input channel (*SYN*_*t*_, arbitrary units) by multiplying *AN*_*t*_ with a fixed synaptic weight vector (*W*) to mimic the neuron’s frequency tuning [*SYN*_*t*_ = W*AN_*t*_]. At the fourth stage (ASYN), the synaptic activity generated adaptation, *A*_*t*_, according to a monotonic adaptation function, *g(x)*. At the fifth stage (A), the adaptation (*A*_*t*_) was subtracted from the synaptic activity (*SYN*_*t*_) to obtain the actual adapted activation of the synapse, *ASYN*_*t*_ = *SYN*_*t*_ − *A*_*t*_. Stages four and five were iterated recursively for each trial, whereby the new adaptation from the current trial was added to the remaining adaptation from previous trials and all adaptation was assumed to decay by a factor, *R*, from trial to trial; *A*_*t*+*1*_ = *(1* − R)*(A_*t*_ + *g(ASYN*_*t*_)). The adaptation for the first trial, *A*_*1*_, was set to zero. At the sixth stage (IN), the synaptic activities of all channels were integrated in order to obtain the total synaptic input, *IN*_*t*_ = 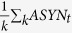 (arbitrary units), where *k* = number of input channels. The seventh and final stage (FR) computed the firing rate of the neuron (*FR*_*t*_) from the integrated synaptic input using a similar threshold-saturating in/out function as used to calculate the auditory-nerve activity [*FR*_*t*_ = *out(IN*_*t*_)].

The shape of the monotonic adaptation function, *g*(*x*) was determined by three parameters, *M*, *m* and *τ* [*g*(*x*) = *α*(*x*)*x; where *α*(*x*) = *m* + (*M* − *m*)*2^(*−x*/*τ*)^], where *M* and *m* represent the initial and final slopes of *g* and *τ* determines the point where the slope is (*M* + *m*)/*2*. Thus, adaptation [*g*(*x*)] was proportional to synaptic activity (*x*), but with a proportionality constant (*α*) that could differ between low and high levels of synaptic activity. We estimated population parameters *M, m* and *τ* for neurons with high and low SSA, respectively (Fig. 8H), by comparing the adaptation in the standard-alone condition (estimated as the difference in firing rate between the deviant- and standard-alone conditions; FR_deviant-alone_ − FR_standard-alone_) with the unadapted synaptic activity (assumed to be given by FR_deviant-alone_). For each individual neuron, the tuning weights (*W*) and parameters of output function *out*() were adjusted to mimic the tuning and threshold of that particular neuron and the parameters of the adaptation function (*M*, *m*, *τ*) were fitted through nonlinear least squares regression of the observed and simulated NRI_*d*_ values across conditions. The mean squared error (MSE) was computed to quantify the difference between the observed and the simulated NRI_*d*_ values.

## Additional Information

**How to cite this article**: Duque, D. *et al.* Neurons in the inferior colliculus of the rat show stimulus-specific adaptation for frequency, but not for intensity. *Sci. Rep.* **6**, 24114; doi: 10.1038/srep24114 (2016).
